# Encapsulation of Trimethine Cyanine in Cucurbit[8]uril: Solution versus Solid‐State Inclusion Behavior

**DOI:** 10.1002/chem.202200185

**Published:** 2022-03-21

**Authors:** Giuseppe Soavi, Alessandro Pedrini, Anjali Devi Das, Francesca Terenziani, Roberta Pinalli, Neal Hickey, Barbara Medagli, Silvano Geremia, Enrico Dalcanale

**Affiliations:** ^1^ Department of Chemistry Life Science and Environmental Sustainability University of Parma Parco Area delle Scienze 17/A 43124 Parma Italy; ^2^ Centre of Excellence in Biocrystallography Department of Chemical and Pharmaceutical Sciences University of Trieste Via L. Giorgieri 1 34127 Trieste Italy

**Keywords:** crystal structure, cucurbit[8]uril, cyanines, host-guest chemistry

## Abstract

Inclusion of polymethine cyanine dyes in the cavity of macrocyclic receptors is an effective strategy to alter their absorption and emission behavior in aqueous solution. In this paper, the effect of the host‐guest interaction between cucurbit[8]uril (CB[8]) and a model trimethine indocyanine (Cy3) on dye spectral properties and aggregation in water is investigated. Solution studies, performed by a combination of spectroscopic and calorimetric techniques, indicate that the addition of CB[8] disrupts Cy3 aggregates, leading to the formation of a 1 : 1 host‐guest complex with an association constant of 1.5×10^6^ M^−1^. At concentrations suitable for NMR experiments, the slow formation of a supramolecular polymer was observed, followed by precipitation. Single crystals X‐ray structure elucidation confirmed the formation of a polymer with 1 : 1 stoichiometry in the solid state.

## Graphical abstract

**The different outcome** of the dye Cy3 inclusion in CB[8] cavity in the solid state and in water has been elucidated via crystal structure determination and NMR, ITC and fluorescence titrations respectively. The initial 1 : 1 complex formed in water evolves spontaneously toward an insoluble linear polymeric structure.

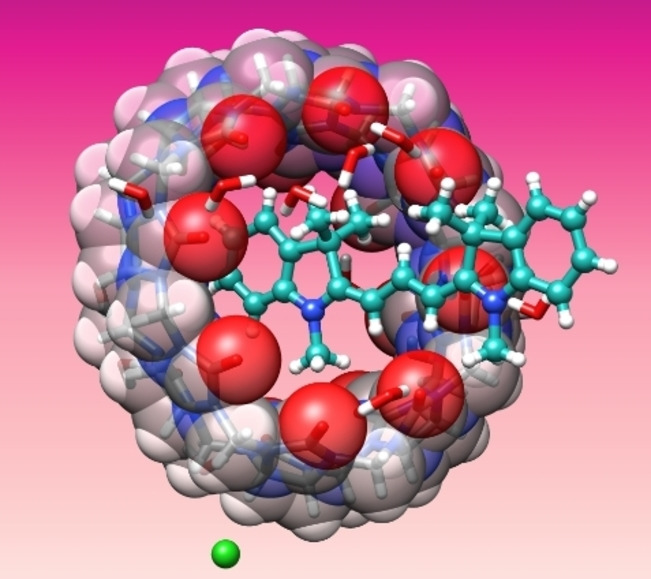

Cyanines are widely used organic fluorescent dyes whose attractiveness is due to their favorable optical properties such as high molar extinction coefficient, suitable fluorescence quantum yield and narrow absorption/emission bands.^[1]^ These molecules belong to the polymethine class of dyes and present a planar, conjugated chain of sp^2^‐hybridized carbon atoms with an odd number of methine groups and an even number of π electrons between the two nitrogen‐containing heterocyclic rings.[^2^, ^3^] According to the number of carbon atoms of the chain, these dyes are usually referred to as monomethine (Cy1), trimethine (Cy3), pentamethine (Cy5), and heptamethine cyanines (Cy7). The tunability of their fluorescence profiles from UV‐vis to near‐infrared (NIR) by changing the polymethine chain length or by inserting functional groups on the heterocycles, together with their outstanding biocompatibility and low toxicity, makes cyanines suitable probes for medicinal and biological imaging.[^4^, ^5^] In the early 1990’s, cyanine dyes with increased water solubility and reduced fluorescence quenching by dye‐dye interactions became commercially available in the succinimidyl ester form. These ready availability and improved properties kick‐started their widespread use as labelling agents for nucleic acids. Being highly sensitive to microenvironmental factors such as hydrogen bonding and viscosity‐dependent C−C bond rotational motion,[^6^, ^7^] cyanines are also promising candidates for the development of molecular fluorescent probes for the visualization of viscosity in living biosystems.^[8]^

Rapid photobleaching and the tendency to form aggregates in aqueous solutions are common drawbacks for the use of these dyes in the above mentioned applications.[^9^, ^10^, ^11^] Encapsulation of fluorescent molecules in nanoparticles or macromolecules can drastically change their local microenvironments, subsequently affecting their stability and spectral properties.^[12]^ In particular, host‐guest complexation by water‐soluble macrocyclic receptors is a powerful tool to fine‐tune the absorption and emission behavior of the dyes.^[13]^ For example, cyclodextrins have been shown to dramatically alter the microenvironment of cyanines, leading to the disruption of aggregates in favor of the formation of dimers and inhibiting their photodegradation.[^14^, ^15^, ^16^] The formation of a rotaxane by the threading of a α‐cyclodextrin by the heptamethine chain of a Cy7 was found to increase both chemical stability and water‐solubility of the dye.^[17]^ In addition, cucurbit[7]uril (CB[7]), a synthetic macrocycle characterized by a toroidal, hydrophobic cavity constituted by seven glycoluril units bound together by 14 methylene bridges, has been demonstrated to effectively complex monomeric Cy1 and Cy3 dyes thereby disrupting their J‐ and H‐aggregates, respectively.[^9^, ^18^] The same macrocycle was exploited to alter the photophysical properties of a different Cy3 guest^[19]^ and a linear cyanine dye (LDP)^[20]^ in aqueous solution. Moreover, Cy3‐conjugated CB[7] (Cy3‐CB[7]) found several application as live cell imaging probe, both alone[^21^, ^22^] and as component of high affinity host‐guest Förster resonance energy transfer (FRET) pairs.[^23^, ^24^, ^25^]

If CB[7] has been well exploited in combination with cyanines, interactions between these dyes and cucurbit[8]uril (CB[8]) are far less investigated. Bearing an additional glycoluril unit (Scheme 1), CB[8] has a larger portal diameter (6.9 Å) and inner cavity volume (479 Å^3^) than CB[7].^[26]^ These features allow for additional binding modes as well as various stoichiometries, ranging from the simplest 1 : 1 binary complexes^[27]^ to discrete (n:n) host:guest oligomers.^[28]^ Depending on the guests structures, 2 : 1,^[29]^ 1 : 2 homoternary,^[30]^ 1 : 1 : 1 heteroternary^[31]^ and 2 : 2 quaternary^[32]^ complexation modes are possible. The binding mode of CB[8] complexes has a strong impact on their spectral properties, in particular on fluorescence emission.^[33]^

**Scheme 1:**
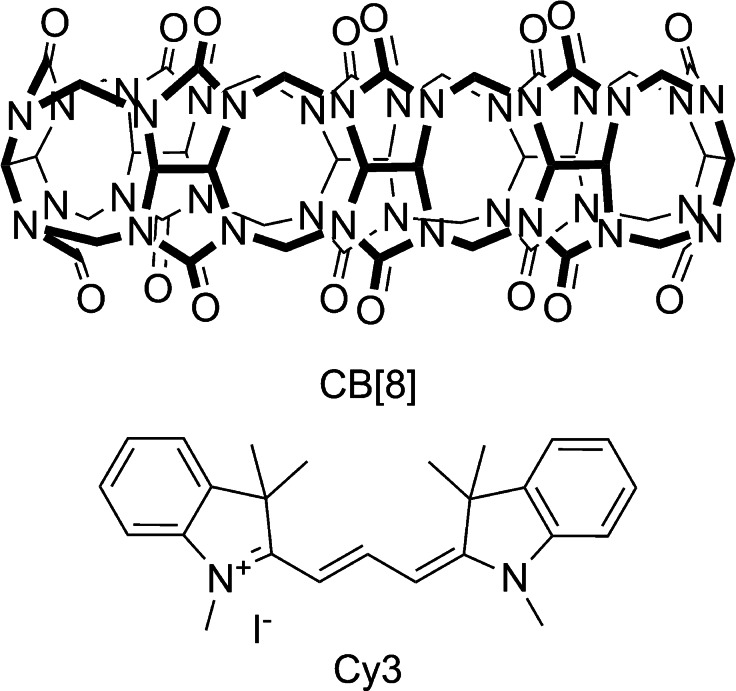
Structures of CB[8] and the cyanine dye Cy3 involved in this study.

Herein, we investigated the interaction of a model indocyanine trimethine dye, namely 1‐methyl‐2‐[3‐(1‐methyl‐3,3‐dimethylindolin‐2‐ylidene)prop‐l‐enyl]‐3,3‐dimethyl‐3*H*‐iodolium iodide (Cy3), with CB[8] (Scheme 1). The dye was synthesized following a two‐step procedure reported in the literature (Scheme S1).^[34]^ Briefly, 1‐Methyl‐2,3,3,–trimethyl‐3H‐indolium iodide was synthesized by refluxing 2,3,3‐trimethyl‐3H‐indole with iodomethane at 130 °C for 6 h. Subsequently, the red product was reacted with triethylorthoformate in pyridine to give dye Cy3 which was purified by recrystallization in ethanol. CB[8] was prepared by acid‐catalyzed condensation of glycoluril with formaldehyde and purified accordingly to a well‐established protocol based on repeated precipitations.^[35]^

The binding interaction between Cy3 and CB[8] was firstly monitored via matrix‐assisted laser desorption ionization time‐of‐flight (MALDI‐TOF) spectrometry and ^1^H NMR spectroscopy in D_2_O. The high‐resolution MALDI‐TOF spectrum of an aqueous solution of the two compounds in stoichiometric ratio evidenced the presence of the binary complex (Figure S1). Figure 1 shows the difference between the ^1^H NMR spectrum of Cy3 at 0.1 mM and the spectra obtained after addition of CB[8]. A sizable upfield shift was observed for the aromatic protons (H_1,2,3,4_) upon addition of 0.4 equiv. of CB[8] (Figure 1b), while slight upfield shifts were also seen for the signals of the polymethine chain (H_7,8_). Therefore, the largest perturbation was observed for the protons in the aromatic region, thus suggesting an asymmetric binding of the longer Cy3 molecule with respect to the depth of the CB[8] cavity. Indeed, symmetric 1 : 1 binding should expose the aromatic protons outside the macro‐ring. On the other hand, it is notable that the spectrum still shows symmetric signals for the Cy3 molecule. This can be rationalized in terms of fast exchange between complexed and uncomplexed Cy3 molecules. Additionally, when the ^1^H NMR spectrum was recorded with one equivalent of CB[8] (Figure 1c), the signals of Cy3 were significantly broadened and were barely visible. A similar absence of sharp signals was also observed by Scherman and co‐workers in other CB[8] complexes.^[36]^ This was attributed to the formation of a supramolecular polymer in solution.

**Figure 1:**
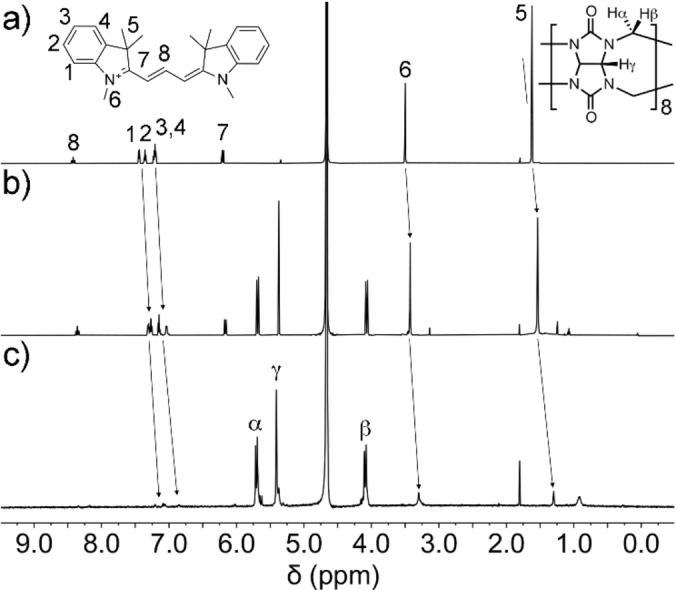
^1^H NMR (600 MHz, D_2_O): addition of increasing amounts of CB[8] to Cy3: (a) Cy3 at 0.1 mM; (b) addition of 0.4 equiv. of CB[8]; (c) addition of 1 eq. of CB[8].

Isothermal titration calorimetry (ITC) experiments were performed to investigate the binding strength, stoichiometry and thermodynamic parameters for the host‐guest complexation of Cy3 by CB[8]. Figure 2 shows a typical ITC titration experiment with Cy3 and CB[8]. A 27 injection experiment was carried out at *T*=25 °C where the cell contains a 0.1 mM aqueous solution of CB[8], and Cy3 is in the syringe at a concentration of 1.0 mM. The observed exothermic binding curve has a sharp inflection point at a molar ratio of one indicating the formation of a 1 : 1 assembly in aqueous solution. A second and less pronounced binding event is present when 0.5 equivalents of guest are added to the host solution, suggesting the coexistence of a 2 : 1 host‐guest complex. The data fitting of the titration with a “two sequential binding sites” model gave an association constant *K*_a,1_=3.9×10^7^ M^−1^ with a Δ*H*_1_ of −5.1 kcal mol^−1^ for the first binding of the guest and a *K*_a,2_ of 1.4×10^5^ M^−1^ plus a Δ*H_2_* of −0.7 kcal mol^−1^ for the interaction with the second host. The corresponding calculated thermodynamic parameters Δ*G*_1_ and *T*Δ*S*_1_ are −10.4 kcal mol^−1^ and 5.3 kcal mol^−1^, respectively, while Δ*G_2_* =−5.6 kcal mol^−1^ and *T*Δ*S*_2_=4.9 kcal mol^−1^, making the two binding processes both enthalpy and entropy driven. These thermodynamic values are in agreement with the displacement of high energy water molecules from the cavity by the guest, which is known to be one of the main driving forces of CB[8] complexation.^[37]^

**Figure 2:**
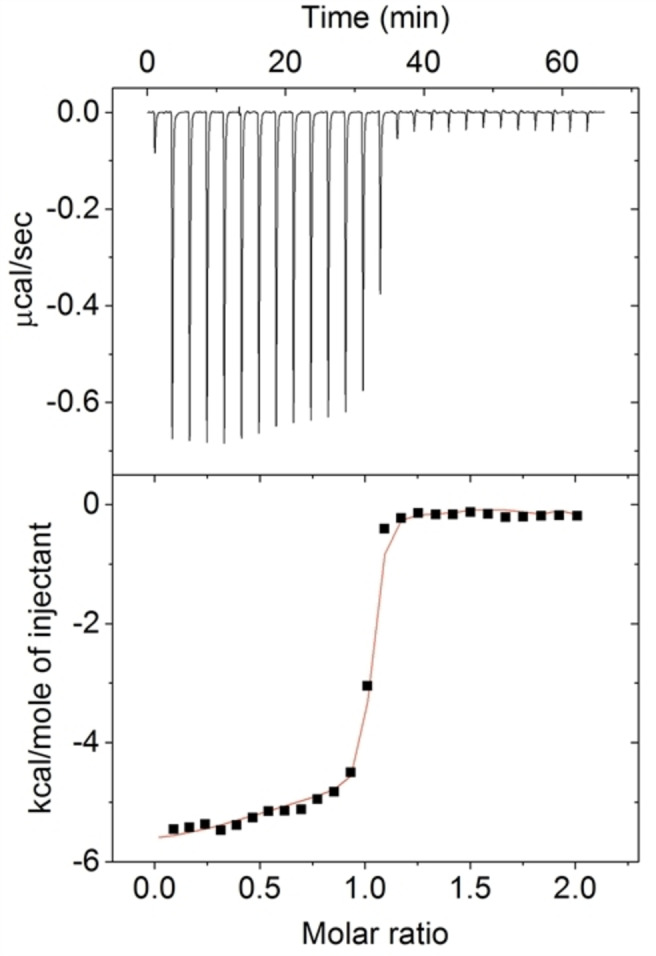
Isothermal calorimetric data obtained in the titration experiment of a CB[8] solution by adding aliquots of the Cy3 guest; [CB[8]]=0.1 mM, [Cy3]=1.0 mM.

The spectroscopic behavior of the dye upon addition of CB[8] was studied in aqueous solution at a concentration of 1 μM to minimize dye self‐aggregation and any effect of inner filter. As shown in Figure 3a, free Cy3 shows two UV absorption maxima, one at 540 nm (main band) and another at 510 nm (vibronic band). Upon addition of CB[8] a red shift is observed in the *λ_max_* along with a decrease in the absorption coefficient. A precise isosbestic point is observed at 545 nm only at low CB[8]/Cy3 ratio, suggesting that at higher CB[8] content the equilibrium is not between two unique species. This spectral shift is in line with a change in the microenvironment of the dye caused by its inclusion in the hydrophobic cavity of CB[8], as already reported in literature for the tetrafluoroborate analogue.^[38]^

**Figure 3:**
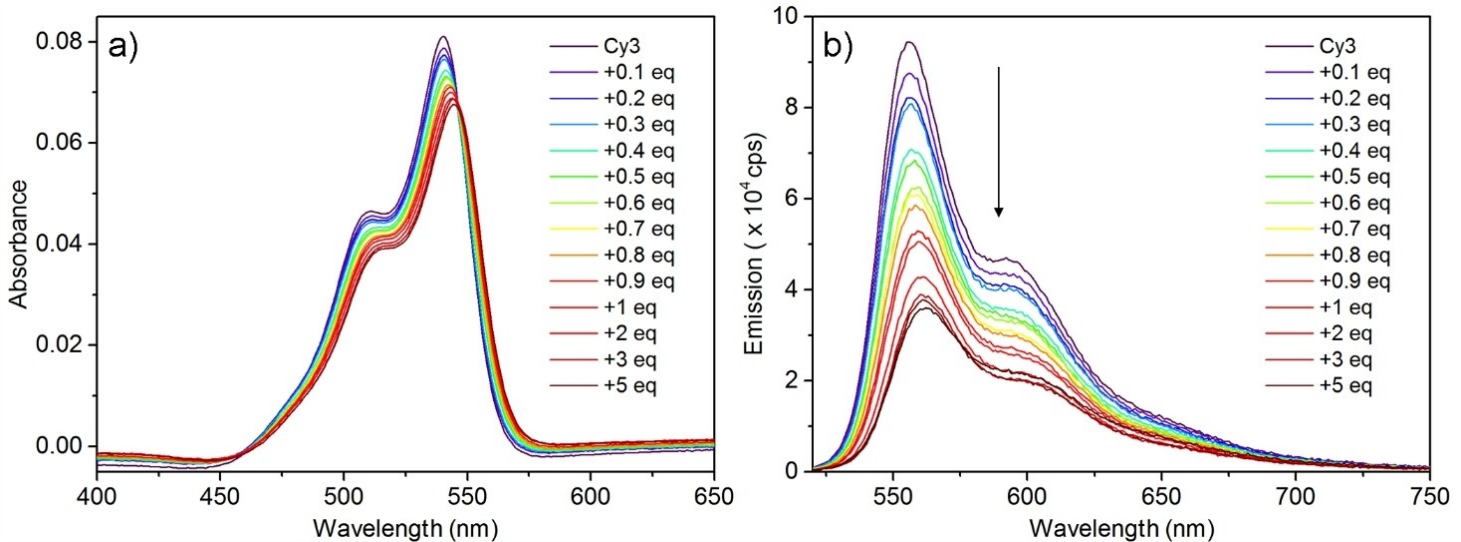
a) UV Visible spectrum of Cy3 (1 μM) in aqueous solution upon addition of CB[8]. (b) Fluorescence spectrum of Cy3 in water (1 μM) upon addition of CB[8], λ_ex_=510 nm.

Fluorescence spectra show a small red shift upon addition of increasing equivalents of CB[8], as well as a significant decrease of the intensity (the fluorescence quantum yield amounts to 1 % free Cy3 and to 0.7 % for the 1 : 1 complex). This last observation is in contrast with the idea of an enhanced emission upon complexation in a restricted environment and deserves more investigation. Fluorescence decays were measured, getting an average fluorescence lifetime of 3.48 ns for free Cy3 in water, and of 4.24 ns for a solution containing the supramolecular 1 : 1 complex (Figure S2). Fluorescence intensity and lifetime data unexpectedly lead to a smaller radiative decay rate for Cy3 in the complex (1.62×10^−3^ ns^−1^) than in water solution (2.99×10^−3^ ns^−1^), consistently with the observed decrease in absorption coefficient upon complexation, while the non‐radiative decay rate stays practically constant (0.287 vs. 0.234 ns^−1^). Since we know that only one Cy3 molecule is present in the complex, the increase of the radiative decay constant (related to transition dipole moment) for free Cy3 in water suggest the presence of aggregates when CB[8] is not present in water. In particular, the results are consistent with slightly superradiant aggregates, which are disaggregated upon complexation with CB[8].

To prove this hypothesis, anisotropy measurements were performed for free Cy3 both in water and in a much more viscous solvent (Figure S3). For free Cy3 in water a fluorescence anisotropy value r=0.26 was found, while the measurement in glycerol gave the fundamental anisotropy value r_0_=0.35. Exploiting the Perrin equation, a hydrodynamic volume of 4.41×10^4^ Å^3^ was estimated for Cy3. Scaling down this value by a factor 1.3,^[39]^ to roughly exclude the solvation shell, we end up with a volume of 3.39×10^4^ Å^3^, corresponding to a sphere of radius 20 Å. This value is comparable with the molecular length, suggesting the presence of oligomeric aggregates in water, despite the low concentration. Therefore, fluorescence anisotropy measurements confirm the presence of small aggregates (not detected, in fact, with DLS) for free Cy3 in water. Cy3 molecules in these aggregates are probably very loosely interacting, as suggested by the small spectral variations with respect to the complex and by the ease of rupture of the aggregates when adding CB[8].

To unambiguously confirm the presence of Cy3 aggregates in water, we performed absorption and fluorescence measurements on solutions at different concentration. Since Cy3 does not have a good solubility in water already at concentrations of the order of 10^−5^ M, we prepared a mother solution and filtered it, to be sure to remove any non‐solubilized compound. The filtered mother solution was then diluted by a factor 8 and by a factor 16. The corresponding spectra (reported in Figure S4), show a decrease of extinction coefficient and of concentration‐normalized emission intensity upon dilution. This strongly supports the formation of aggregates, favored in more concentrated solutions, and the superradiant nature of the aggregates (related to increased transition dipole moment).

Under the premise of dye aggregation, the *K*_a_ estimated by ITC is only an apparent binding constant. We therefore used the fluorescence titration (at much lower Cy3 concentration with respect to the ITC experiment) to obtain a more realistic (albeit still apparent) *K*_a_. The fluorescence data could be fitted through the Benesi‐Hildebrand equation, and confirmed a 1 : 1 host:guest complex, with a *K*_a_=1.5×10^6^ M^‐1^ (see Figure S5 and Supporting Information for details). Interestingly, the major effect of complexation on fluorescence intensity seems to be played by the rupture of aggregates upon the formation of the complex.

While most complexes of CB[8] show a much higher solubility in water than the CB[8] itself, it was surprising to see that during solution studies of binding of the dye Cy3 to CB[8], upon the addition of one equivalent of CB[8] over the course of a few hours the complex started to precipitate as bright purple needle‐shaped crystals. In order to more thoroughly investigate the arrangement of Cy3 in the cavity of CB[8], crystals suitable for X‐ray diffraction (XRD) were grown in water. Diffraction data from small twinned crystals was collected using synchrotron radiation at 100 K. The crystal belongs to the polar orthorhombic Pna2_1_ space group. The solid state structure shows that the asymmetric unit contains one CB[8] macrocycle, one trans Cy3 cation with its chloride counterion and a total of 13 co‐crystallized water molecules (Figure 4a). Therefore, the crystal has a 1 : 1 host‐guest ratio, as observed in solution by spectroscopic and ITC experiments. The aromatic indolinene terminals of the dye are located inside the hydrophobic cavities of CB[8] (Figure 4b). The architecture of the host‐guest interactions furnishes a rationale for the upfield shifts observed for the aromatic protons in the ^1^H NMR investigation.

**Figure 4:**
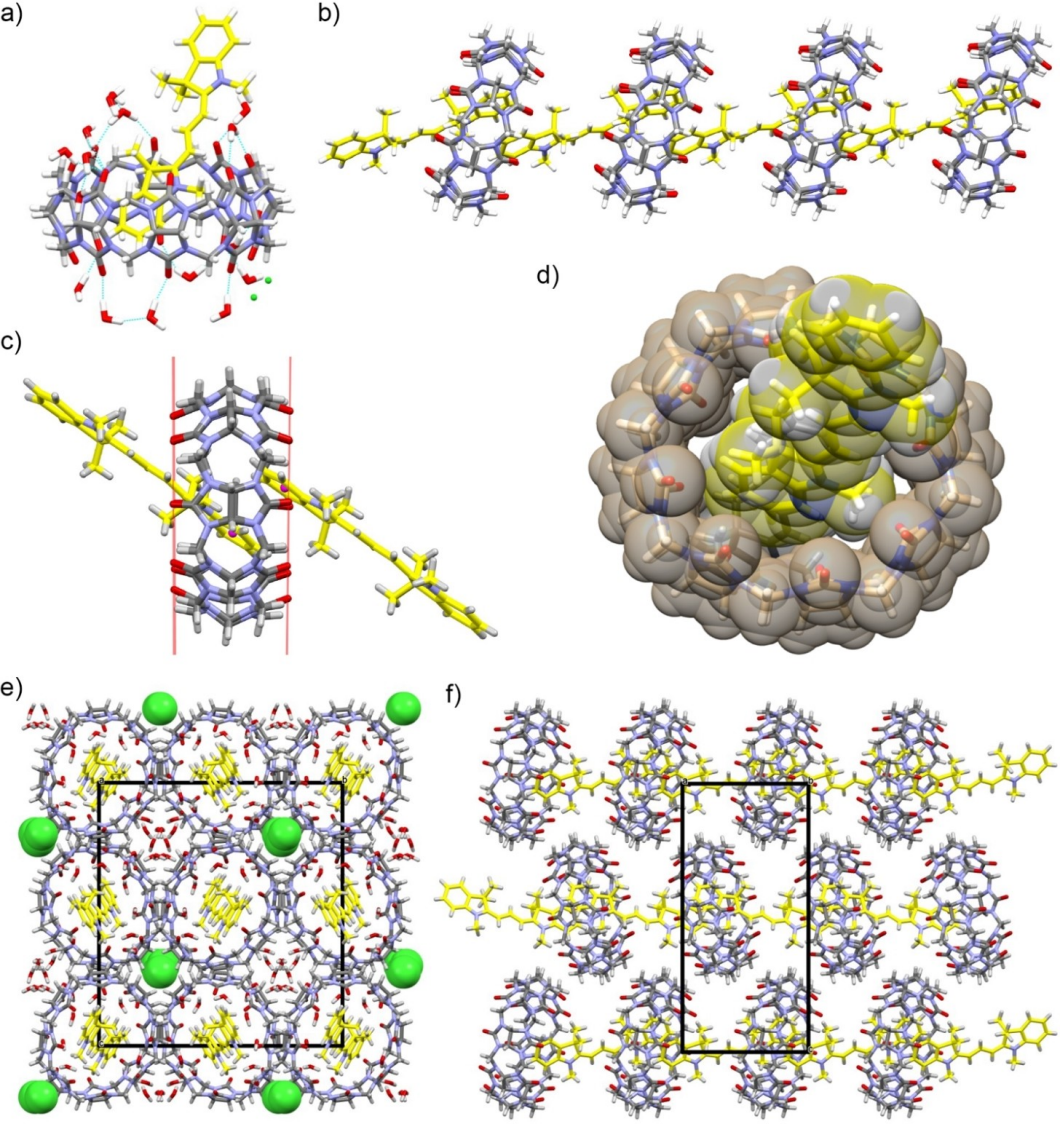
(a) Stick representation in cpk colours of the asymmetric unit of CB[8]•Cy3 complex. Carbon atoms of the Cy3 dye are evidenced in yellow. (b) Linear supramolecular polymeric chain assembly formed by CB[8] and Cy3 units. (c) Each CB[8] unit hosts a couple of *π*‐*π* stacked Cy3 guests. The barycenters of the aromatic ring of the dyes (violet spheres) are differently inserted in the hydrophobic cavity of CB[8] delimitated by the oxygen portal planes (red lines). (d) Space fill representation of the cavity of CB[8] unit completely filled by the aromatic terminals of the Cy3 dyes. (e) Checkerboard pattern of the antiparallel polymeric chains viewed along the crystallographic *a* axes. The chloride counterions (green vdW spheres) are located in alternate water channels. (f) Highly distorted herringbone motive of a single layer of CB[8] units viewed along the crystallographic b axes. The CB[8] macrorings of antiparallel supramolecular polymeric chains are tilted by about 30°.

In fact, the crystal packing shows that both aromatic terminals of an indolenine are hosted by two adjacent CB[8] units (Figure 4b) and that each CB[8] hosts the aromatic terminals of two indolenine guests (Figure 4c). This leads to the formation of a linear supramolecular polymer, as shown in Figure 4b, generated by the periodic crystallographic translation of 12.871 Å along the *a* axes. One aromatic ring of the dye is almost exactly centered in the hydrophobic cavity, with distances of its barycenter from the mean planes of the two CB[8] oxygen portals of 3.16 Å for the entry portal and 2.98 Å for the second portal (Figure 4c). The second aromatic ring is less deeply inserted in the hydrophobic cavity of an adjacent CB[8], with distances of 0.22 and 5.96 Å from the entry and second portals, respectively (Figure 4c). The aromatic plane of the dye is tilted by about 56° with respect to the parallel oxygen portal planes of the polymeric CB[8] chain. The two parallel phenyl rings hosted inside the same CB[8] form a weak *π*‐*π* stacking interaction at a distance of about 3.5 Å (Figure 4c). The asymmetric interaction of the two dyes with the CB[8] produces an asymmetric deformation of the host. The CB[8] entry portal of the more deeply inserted Cy3 is more ovalized^[40]^ with respect to the entry portal of the less inserted one. The difference between the minor and the major axes of the ellipsoidal portals are 1.41 and 1.09 Å, respectively. The aromatic terminals of the dye molecules completely fill the hydrophobic cavity of CB[8] units (Figure 4d) and the cocrystallized water molecules are all external to the cavity.

The crystal packing shows that each linear polymeric chain packs against four other chains oriented in antiparallel fashion giving a checkerboard pattern when viewed along the crystallographic *a* axes (Figure 4e). Therefore, the molecules are arranged in two orthogonally oriented close packed layers parallel to the *ab* and *ac* crystallographic planes (Figure 4f). In these layers, each CB[8] of the supramolecular polymer is offset by half of the a axis length and tilted by about 30° with respect to neighboring antiparallel chains. This results in a highly distorted herringbone motive (Figure 4f). The charges of the dyes are counterbalanced by chloride anions located in alternate water channels bordered by four polymeric chains (Figure 4e).

This crystal packing is similar to that observed for 1 : 2 host‐guest complex between CB[8] and 4‐(4‐aminophenyl)‐N‐methylpyridinium^[41]^ and for the highly disordered polymeric 1 : 1 host‐guest complex between CB[8] and N‐(4‐(phenylazo)benzyl)‐N’‐methyl‐4,4’‐bipyridinium.^[36]^ It is also interesting to note the structural similarity of the columnar arrangement of the Cy3‐CB[8] complex with the reported nanotubular framework constituted by pure CB[8], obtained in the presence of a molecular chaperone,^[42]^ which results in a slight increase (about 0.15 Å) in the distance between consecutive CB[8] molecules.

In conclusion, we investigated the interaction of a indocyanine trimethine dye, Cy3, with cucurbit[8]uril, CB[8], by MS, NMR, ITC, UV‐Vis absorption and fluorescence spectroscopy and single crystal X‐ray diffraction. The solution studies indicate the formation of a 1 : 1 complex of Cy3 and CB[8]. The ITC measurements reveal that the complexation is an enthalpically and entropically driven process, with an apparent association constant of 1.5×10^6^ M^−1^, obtained via fluorescence titration. The optical spectroscopic investigation suggests the presence of Cy3 aggregates in water which are disrupted by addition of CB[8]. The ^1^H NMR spectra confirm the complexation process and evidence a slow formation of a supramolecular polymer with subsequent precipitation after a few hours. The X‐ray structure obtained from single crystals confirms the formation of a polymer with 1 : 1 stoichiometry in the solid state. Both aromatic terminals of Cy3 are hosted by two adjacent CB[8] units and each CB[8] hosts the aromatic terminals of two Cy3 guests, thereby forming linear supramolecular polymeric chains. The columnar arrangement of the Cy3⋅CB[8] complex represents an interesting nanotubular framework with incorporated dye molecules.

## Crystallographic Details

Deposition Number(s) 2092004 contain(s) the supplementary crystallographic data for this paper. These data are provided free of charge by the joint Cambridge Crystallographic Data Centre and Fachinformationszentrum Karlsruhe Access Structures service.

## Conflict of interest

The authors declare no conflict of interest.

## Supporting information

As a service to our authors and readers, this journal provides supporting information supplied by the authors. Such materials are peer reviewed and may be re‐organized for online delivery, but are not copy‐edited or typeset. Technical support issues arising from supporting information (other than missing files) should be addressed to the authors.

Supporting Information

## Data Availability

The data that support the findings of this study are available from the corresponding author upon reasonable request.
