# Supplementary material for: Encapsulation of Trimethine Cyanine in Cucurbit[8]uril: Solution versus Solid‐State Inclusion Behavior
Source: Chemistry. 2022 Mar 21;28(23):e202200185. doi: 10.1002/chem.202200185 (PMC9313864; doi:10.1002/chem.202200185)
Supplement: Supplementary file 1 — Supporting Information [file CHEM-28-0-s001.pdf]

Chemistry–A European Journal

Supporting Information

Encapsulation of Trimethine Cyanine in Cucurbit[8]uril: Solution versus Solid-State Inclusion Behavior

Giuseppe Soavi, Alessandro Pedrini, Anjali Devi Das, Francesca Terenziani, Roberta Pinalli, Neal Hickey, Barbara Medagli, Silvano Geremia,* and Enrico Dalcanale*

1 General experimental methods

Synthesis

Unless stated otherwise, all reactions were carried out under strictly anhydrous conditions under Ar atmosphere. All solvents were dried and distilled using standard procedures. All commercially obtained reagents were used as received unless otherwise specified. Silica column chromatography was performed using silica gel (Fluka 230 – 400 mesh).

NMR spectroscopy

NMR spectra were collected on Bruker Avance 300 (300 MHz) and JEOL ECZ600R (600 MHz) spectrometer at 25 °C. ^{1}H and ^{13}C NMR chemical shifts (δ) are given in part per million (ppm) and calibrated to either residual solvent signal. NMR data are reported in the following format: chemical shift (multiplicity (s = singlet, d = doublet, t = triplet, q = quartet, dd = doublet of doublets, m = multiplet), coupling constants (Hz), integration).

Mass spectrometry

Electrospray ionization mass spectrometry (ESI-MS) experiments were performed using a Waters ZMD spectrometer equipped with an electrospray interface. High-resolution MALDI-TOF was performed using an AB SCIEX MALDI TOF-TOF 4800 Plus (matrix: α-cyano-4-hydroxycinnamic acid).

UV-Vis absorption and fluorescence spectroscopy

Optical measurements were performed on Thermo Scientific™ Evolution™ 260 Bio UV-Vis spectrophotometer and on an Edinburgh FLS1000 spectrofluorometer.

2 Experimental procedures

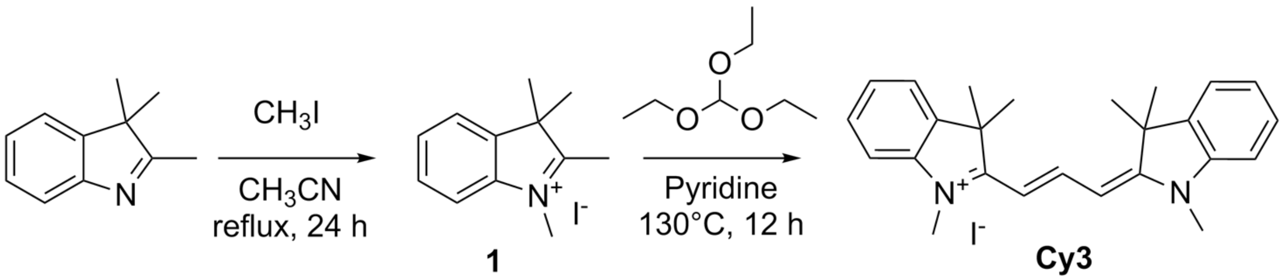

Scheme S1. Synthesis of cy3.

2.1 Synthesis of 1,2,3,3-tetramethyl-3H-indol-1-ium iodide (1)

2,3,3-Trimethyl-3H-indole (4.77 g, 30 mmol) was dissolved in a mixture of methyl iodide (24.11 mL, 300 mmol) and acetonitrile (60 mL) under Ar. The solution was refluxed for 24 h, then allowed to cool to room temperature and the resulting solid was collected by filtration. The solid was washed with acetone and recrystallized with EtOH to give compound **1** as yellow crystalline compound (4.25 g). Yield, 45%.

^{1}H NMR (300 MHz, DMSO-d_6): δ (ppm) = 7.93-7.90 (m, 1H), 7.85-7.82 (m, 1H), 7.64-7.61 (m, 2H), 3.98 (s, 3H), 2.78 (s, 3H), 1.53 (s, 6H);

^{13}C NMR (75 MHz, DMSO-d_6): δ (ppm) = 196.4, 142.6, 142.1, 129.8, 129.3, 123.8, 115.6, 54.5, 35.3, 22.2, 14.7;

ESI-MS: m/z calcd. for $C_{12}H_{16}N^+$ $[M]^+$: 174.13, found 174.25.

2.2 Synthesis of 1-methyl-2-[3-(1-methyl-3,3-dimethylindolin-2-ylidene)prop-l-enyl]-3,3-dimethyl-3*H*-iodolium iodide (Cy3)

Compound **1** (0.63 g, 4 mmol) was refluxed with triethylorthoformate (0.6 mL, 8 mmol) in 3 mL of pyridine at 120 °C for 1 h. The reaction mixture was then cooled to room temperature and excess diethyl ether was carefully added to the solution to precipitate the product. The crude dye was recrystallized from ethanol to give the product with a 58% yield.

^{1}H NMR (300 MHz, DMSO-d_6): δ (ppm) = 8.34 (t, J = 13.6 Hz, 1H), 7.64 (d, J = 7.4 Hz, 2H), 7.46 (m, 4H), 7.29 (m, 2H), 6.45 (d, J = 13.6 Hz, 2H), 3.65 (s, 6H), 1.69 (s, 12H);

^{13}C NMR (75 MHz, DMSO-d_6): δ (ppm) = 174.8, 149.9, 143.3, 141.0, 129.1, 125.8, 123.0, 112.1, 103.2, 49.3, 32.0, 27.7;

ESI-MS: m/z calcd. for $C_{25}H_{29}N_2^+$ $[M]^+$: 357.23, found 357.95.

3 Mass spectrometry

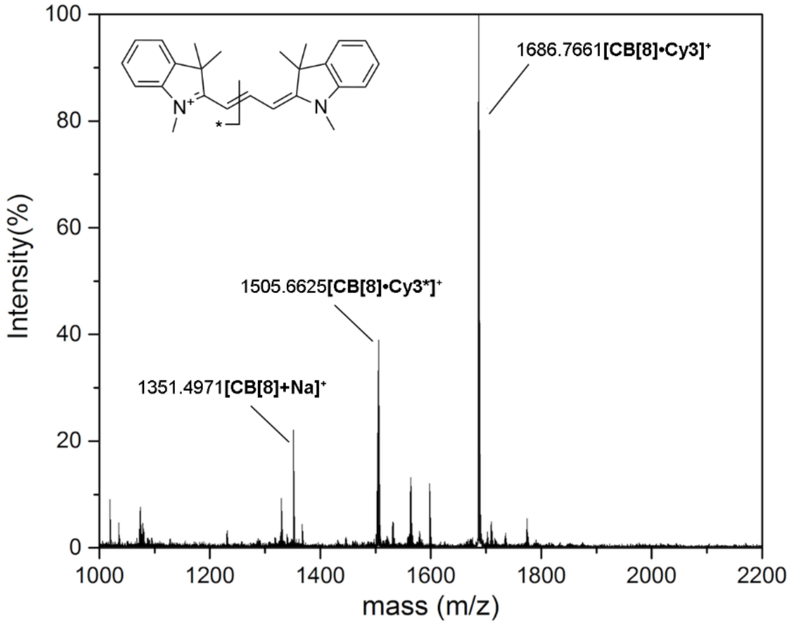

Figure S1. High-resolution MALDI-TOF spectrum (expanded 1000-2200 m/z region) of CB[8]•Cy3.

4 Spectroscopic characterization

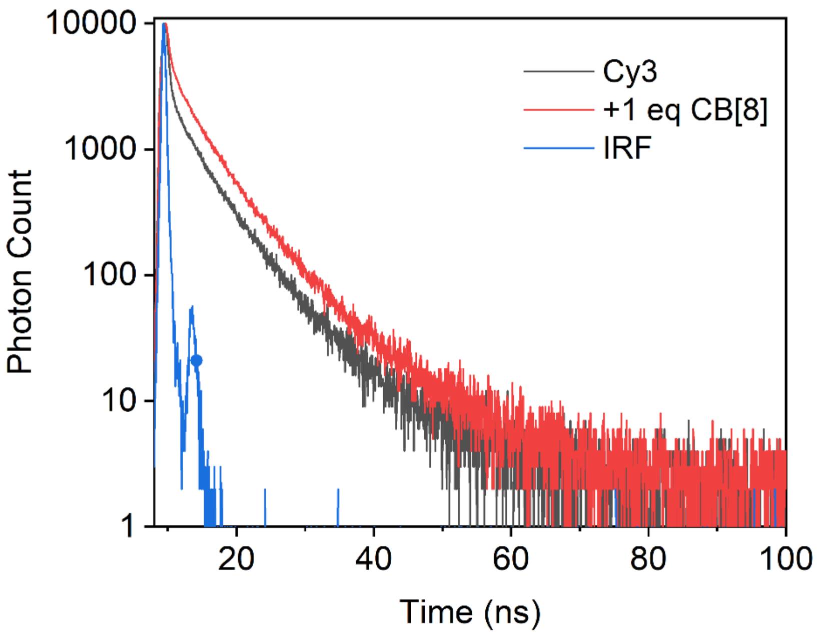

Figure S2 Fluorescence decay traces of free Cy3 (black) and Cy3•CB[8] complex (red) in water.

Table S1. Tri-exponential fit of the fluorescence decay of free Cy3 and of the 1:1 Cy3•CB[8] complex.

	**τ_1 (ns) [f_1 (%)]**	**τ_2 (ns) [f_1 (%)]**	**τ_3 (ns) [f_1 (%)]**	**Average lifetime (ns)**
**Free Cy3**	0.239 [33.473]	2.806 [29.892]	6.911 [36.635]	3.48
**Cy3 +1 eq CB[8]**	0.397 [21.266]	3.435 [46.476]	8.044 [32.258]	4.24

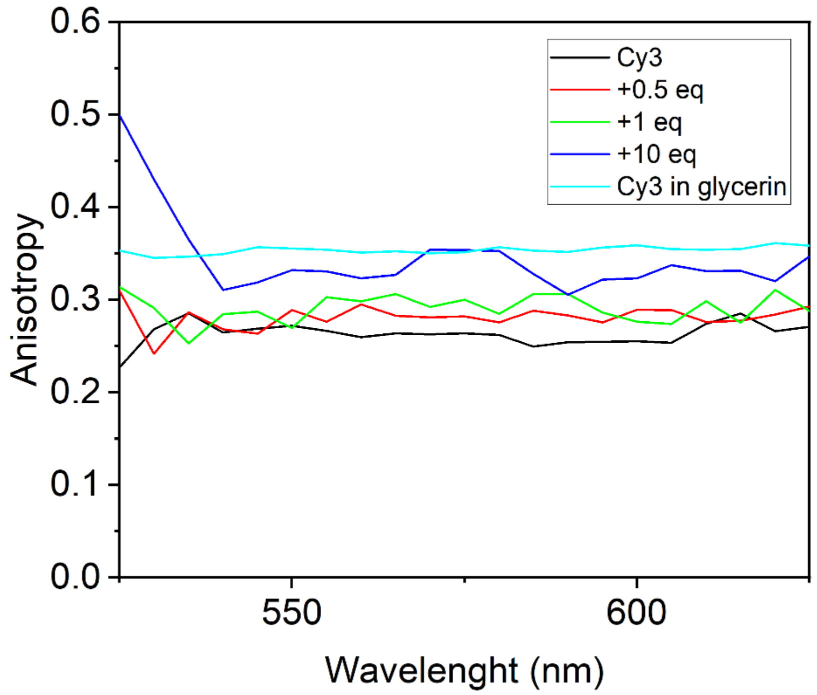

Figure S3 Anisotropy traces for Cy3 (black), Cy3 +0.5 eq CB[8] (red), Cy3 +1 eq CB[8] (green), Cy3 +10 eq CB[8] (blu) in water and anisotropy trace for Cy3 (cyano) in glycerin.

4.1 Hydrodynamic volume evaluation

To assess the existence of aggregates, the rotational correlation time is calculated through the Perrin equation[1]:

$$r = \frac{r_0}{1 + (\tau/\theta)}$$

where r is the anisotropy of Cy3 in water, r_0 is the fundamental anisotropy measured for a solution of Cy3 in glycerol, τ is the fluorescence lifetime and θ is the rotational correlation time. The calculated value for θ is 9.57 ns.

The rotational correlation time, θ, is proportional to the molecular volume[1]:

$$\theta = \frac{\eta V}{RT}$$

where η is the viscosity of the solvent, R is the gas constant and T is the absolute temperature.

4.2 Fluorescence quantum yield

To evaluate the fluorescence quantum yield for the guest and for the solution containing the guest and 1 equivalent of host, the following equation is used[1]:

$$Q = Q_r \frac{I}{I_r} \frac{OD_r}{OD} \frac{n^2}{n_r^2}$$

where Q is the quantum yield, I is the integrated emission intensity, OD is the optical density and n is the refractive index. The subscript "*r*" denotes the reference, namely Rhodamine 101 in ethanol, whose Q_r is known.

4.3 k_r and k_{nr} evaluation

To assess the radiative and the non-radiative decay rates, starting from the quantum yield and the fluorescence lifetime, the following two equations were used[1]:

$$k_r = \frac{Q}{\tau}$$

$$k_{nr} = \tau^{-1} - k_r$$

4.4 Concentration-dependent spectra

A mother solution of Cy3 in water was prepared (nominal concentration on the order of 10^{-5} M) and filtered, to be sure to remove any non-solubilized compound. The filtered mother solution was then diluted by a factor 8 and by a factor 16.

A micro-cuvette of 1.5×1.5 mm path was used for fluorescence measurements, to reduce inner filter effects. For the mother solution, inner filter effects were non-negligible though, therefore the corresponding emission spectrum has been corrected according to the following equation[1]:

$$I_{\text{corr}}(\lambda_{\text{em}}) = I(\lambda_{\text{em}}) \times 10^{[A(\lambda_{\text{exc}})+A(\lambda_{\text{em}})]/2}$$

where $I(\lambda_{\text{em}})$ and $I_{\text{corr}}(\lambda_{\text{em}})$ are the measured and the corrected emission spectra, respectively, $A(\lambda_{\text{exc}})$ and $A(\lambda_{\text{em}})$ are the absorbance values at the excitation and emission wavelength, respectively. The absorbance values are referred to a 1.5 mm path length (as used in fluorescence).

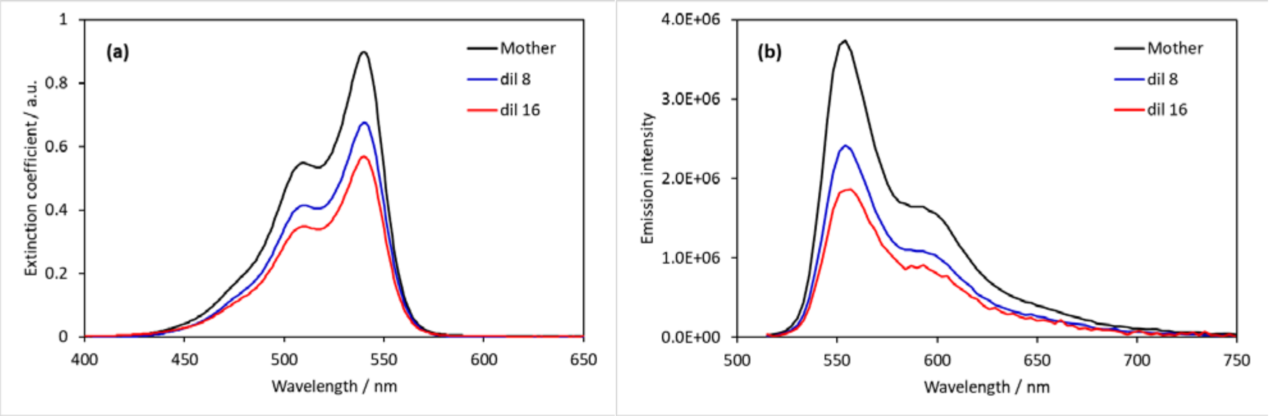

Figure S4 (a) Absorption spectra of Cy3 water solutions having different concentration: the spectra of diluted solutions were divided by the corresponding dilution factor, to retrieve spectra proportional to the molar extinction coefficient. (b) Fluorescence spectra of Cy3 water solutions having different concentration: the spectra were corrected for inner filter effects (significant only for the mother solution); each spectrum was then divided by the absorbance of the corresponding solution at the excitation wavelength.

4.5 Evaluation of the association constant by optical data

To evaluate the association constant (K_a) by fluorescence data, the Benesi-Hildebrand equation was used:

$$\frac{1}{I - I_0} = \frac{1}{K_a(I_{max} - I_0)[L]^n} + \frac{1}{I_{max} - I_0}$$

where $[L]$ is the host concentration, I_0 is the emission intensity in absence of host, I_{max} is the emission intensity at concentration of host beyond which the emission does not change significantly any more (1 eq in our case) and I is the emission intensity for every added equivalent of host, n is the number of host molecules which complex the guest.

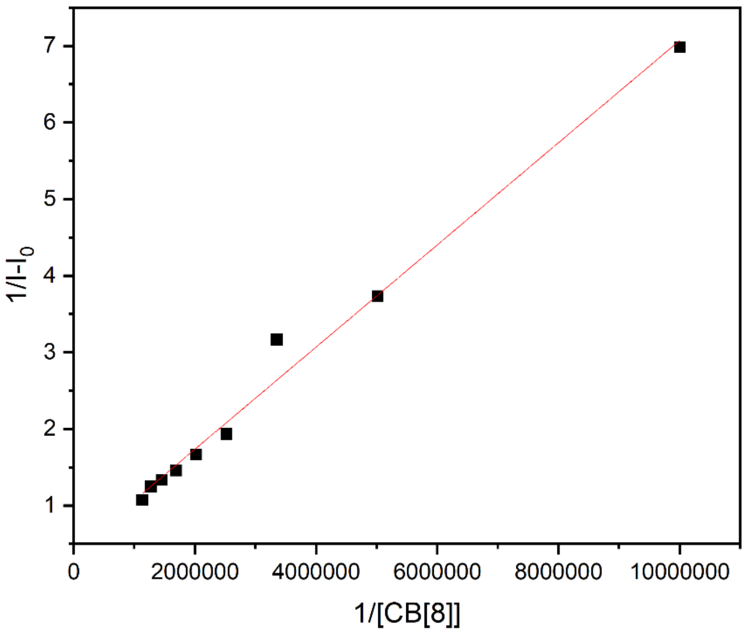

Figure S5 Fitting of data to obtain the association constant. A linear equation well fitted the points, whose equation is y=6.667E-7x+0.4043 (R^2= 0.9887).

1 J.R. Lakowicz *Principles of Fluorescence Spectroscopy*, Third Edition

5 X-ray diffraction from single crystals

Bright purple needle needle-shaped single crystals suitable for an X-ray investigation were obtained by recrystallization in water of the **CB[8]·Cy3** precipitate. Data collection was carried out at the XRD1 beamline of the Elettra synchrotron (Trieste, Italy), employing the rotating-crystal method with a Dectris Pilatus 2M area detector. Single crystals were dipped in paratone cryoprotectant, mounted on a nylon loop and flash-frozen under a nitrogen stream at 100 K. Diffraction data were indexed and integrated using the XDS package,[1] while scaling was carried out with XSCALE.[2] Structures was solved using the SHELXT[3] program and structure refinement was performed with SHELXL-18/3,[4] operating through the WinGX GUI,[5] by full-matrix least-squares (FMLS) methods on F^2. Non-hydrogen atoms were refined anisotropically, with the exception of some water molecules with occupancy factor less of equal to 0.5. Due to the low reflection/parameter ratio the ISOR and SIMU cards were used in the refinement. Hydrogen atoms were added at the calculated positions and refined using the riding model. Crystallographic data and refinement details are reported in Table S2.

The asymmetric unit of the orthorhombic crystals ($Pna2_1$ space group) of **CB[8]·Cy3** (Figure S6) contains one crystallographically independent CB[8] molecule, one Cy3 ion, one chloride ion split in two close positions (0.75/0.25 occupancy factors) and a total of 13 co-crystallized water molecules refined in 17 sites. The structure was refined as a 3-component non-merohedral twin. The twin laws (−1, 0, 0; 0, 0.078, 0.922; 0, 1.078, -0.078), which corresponds to two-fold axes about the [0 5 4] direct lattice direction and (−1, 0, 0; 0, 0.078, -0.922; 0, -1.078, -0.078), which corresponds to two-fold axes about the [0 5 -4] direct lattice direction (Figure S6), were detected with PLATON TwinRotMat. The fraction of overlapped reflections was calculated as about 48% for both components. The refinement as a 3-component twin with HKLF 5 card significantly reduced the R-factor. The R1 factor decreased by 0.060, with refined BASF factors of 0.17 for both components.

1. Kabsch, W. XDS. *Acta Crystallogr. Sect. D Biol. Crystallogr.* **66**, 125–132 (2010).

2. Kabsch, W. Integration, scaling, space-group assignment and post-refinement. *Acta Crystallogr. Sect. D Biol. Crystallogr.* **66**, 133–144 (2010).
3. Sheldrick, G. M. SHELXT - Integrated space-group and crystal-structure determination. *Acta Crystallogr. Sect. A Found. Crystallogr.* **71**, 3–8 (2015).
4. Sheldrick, G.M. Crystal structure refinement with SHELXL. *Acta Crystallogr.* **C71**, 3–8 (2015).
5. Farrugia, L. J. WinGX and ORTEP for Windows: An update. *J. Appl. Crystallogr.* **45**, 849–854 (2012).

Table S2. Crystal data and structure refinement for **CB[8]·Cy3**

	**CB[8]·Cy3**
Empirical formula	$C_{48}H_{48}N_{32}O_{16}$, $C_{25}H_{29}N_2^+$, Cl^-, $13H_2O$
Formula weight	1956.34
Temperature (K)	100(2)
Wavelength (Å)	0.7
Crystal system	Orthorhombic
Space group	P na2_1
Unit cell dimensions (Å)	a = 12.871(15)
	b = 25.367(18)
	c = 27.426(17)
Volume (Å^3)	8955(13)
Z	4
ρ_{calcd} (g/cm^3)	1.451
μ (mm^{-1})	0.118
F(000)	4112
Reflections collected	35107
Indep. Reflections	11113
restraints/parameters	1646/1252
GooF	1.174
Final R indices [I>2σ(I)]	R_1 = 0.1165
	wR_2 = 0.3058
R indices (all data)	R_1 = 0.1581
	wR_2 = 0.3473
CCDC code	2092004

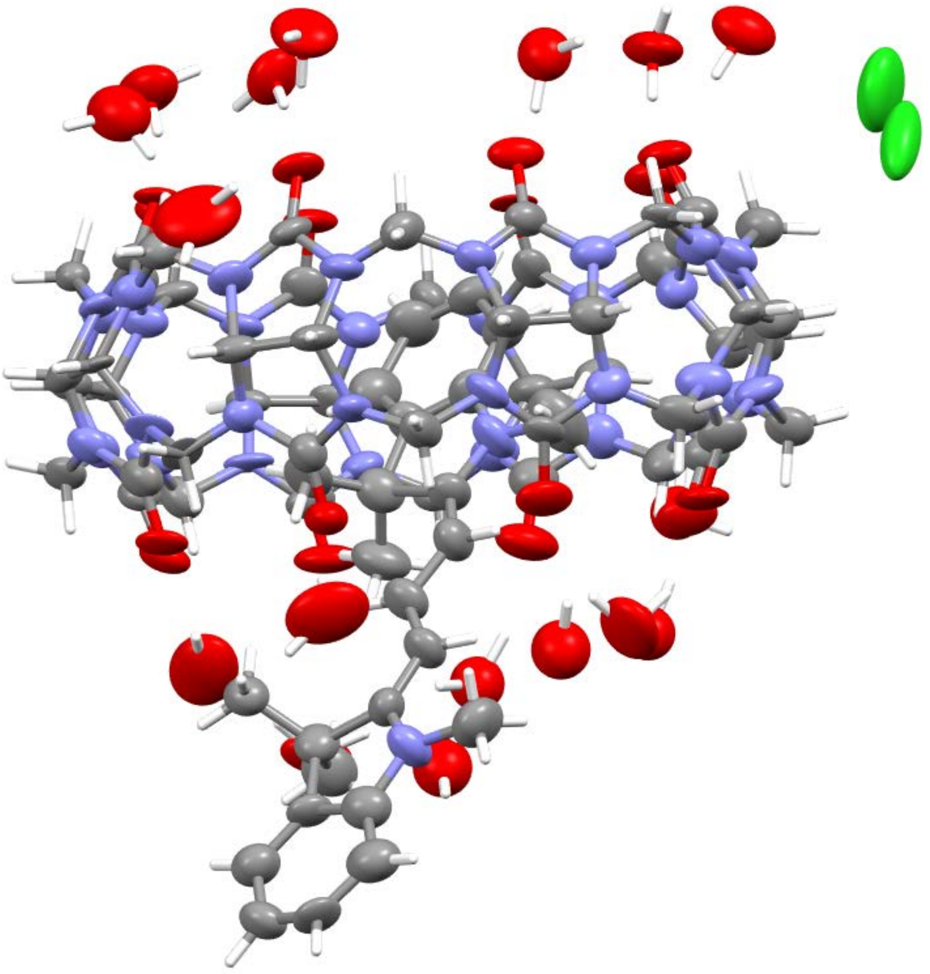

Figure S6. Asymmetric unit of the **CB[8]·Cy3**. Ellipsoids in CPK colors at 50% probability. Hydrogen atoms represented in sticks.
